# Evaluation of superficial xenograft volume estimation by ultrasound and caliper against MRI in a longitudinal pre-clinical radiotherapeutic setting

**DOI:** 10.1371/journal.pone.0307558

**Published:** 2024-07-25

**Authors:** Daniel Roth, Marcella Safi, Oskar Vilhelmsson Timmermand, Evangelia Sereti, Malwina Molendowska, Michael Gottschalk, Anders Bjartell, Crister Ceberg, Filip Szczepankiewicz, Joanna Strand

**Affiliations:** 1 Medical Radiation Physics, Lund, Lund University, Lund, Sweden; 2 Department of Clinical Sciences Lund, Oncology, Lund University, Lund, Sweden; 3 Dept of Translational Medicine, Medical Faculty, Lund University, Lund, Sweden; 4 Lund University Bioimaging Centre, Faculty of Medicine, Lund University, Lund, Sweden; 5 Department of Hematology, Oncology, Radiation Physics, Skåne University Hospital, Lund University, Lund, Sweden; Al-Nahrain University, IRAQ

## Abstract

**Background:**

Accurate tumor volume estimation is important for evaluating the response to radionuclide therapy and external beam radiotherapy as well as to other pharmaceuticals. A common method for monitoring the growth of subcutaneous tumors in pre-clinical models and assessing the treatment response is to measure the tumor length and width by external calipers to estimate its volume. This procedure relies on an assumption of a spheroidal tumor shape wherein the tumor depth equals the width and can yield considerably inaccuracies. Ultrasound imaging is a non-invasive technique that can measure all three axes of the tumor and might be an alternative to caliper measurement with potentially greater accuracy and comparable ease-of-use and throughput. Both 2D and 3D ultrasound imaging are possible, the former offering short scan times without the need for anesthesia and heating—valuable factors for longitudinal studies in large animal cohorts. Nevertheless, tumor volume estimation accuracy by 2D ultrasound imaging has seen limited investigation. In this study we have evaluated the accuracy of tumor volume estimation by caliper and 2D ultrasound with comparisons to reference measurements by magnetic resonance imaging (MRI) in a pre-clinical model of prostate cancer treated with either external beam radiotherapy, radionuclide therapy, or no treatment.

**Results:**

Tumor volumes were measured longitudinally in 29 mice by caliper, ultrasound, and MRI before and after external beam radiotherapy, [^177^Lu]Lu-PSMA-617 radionuclide therapy, or no treatment. Caliper measurements had a marked bias, overestimating the tumor volumes by a median of 150% compared to MRI. Ultrasound measurements were markedly more accurate, with a median bias of -21% compared to MRI.

**Conclusion:**

Ultrasound imaging is a reliable and accurate method for tumor volume estimation in pre-clinical models of radiotherapy, whereas caliper measurements are prone to overestimation.

## Introduction

Pre-clinical models for the evaluation of response to radiotherapy include subcutaneously inoculated xenografts where a decrease or indolence in tumor growth indicates a successful therapy. The routine method for measuring the volume of palpable subcutaneous tumors is by caliper directly on the skin of the animals. For instance, Rodallec et al. reported that calipers were employed in 88.4% of the studies on mice bearing breast cancer xenografts in 2019 [1]. Assuming that the tumor has the shape of an axisymmetric ellipsoid, its length and width can be used to estimate its volume via a simple formula wherein the depth (third axis) is assumed to be equal to the width. The drawback of this method is that tumors frequently have shapes that deviate considerably from an axisymmetric ellipsoid, which leads to inaccurate volume estimates [2]. Furthermore, radiotherapy may alter the shape of the tumor by causing edema, swelling, and necrosis. External beam radiotherapy (EBRT) can also lead to cutaneous inflammation which causes the skin to swell, potentially inflating the estimation error. Finally, epidermis, adipose tissue, and fur may introduce further errors and variability to the estimation. By avoiding the inaccuracy of caliper-based volume estimation, more efficient and reliable experiments can be conducted in the form of more truthful results and conclusions and experiments can be achieved that require fewer animals and less infrastructure to support them.

For treatments where the therapeutic effect stems from radiation damage, accurate dosimetry is pivotal, both for estimating the treatment outcome and for computing absorbed dose-response relationships. In radionuclide therapy, the absorbed dose is commonly calculated following the Medical Internal Radiation Dose (MIRD) formalism [3, 4]. In this formalism, the absorbed dose is calculated using tabulated S-values which summarize the radiation transport and its absorption in a target region for a geometry and radionuclide of interest. S-values depend on the size and mass of the target region [5] and accurate volume estimation is therefore important to enable accurate dosimetry. Accurate size-assessment is particularly important for tumors, as these can often present a large range of shapes and sizes.

The use of ultrasound in pre-clinical research has increased over the last 20 years, as systems capable of resolving the small scales and rapid heart-rates of small animals have developed [6]. The advantages of ultrasound are that it is relatively inexpensive, offers short measurement-times, is non-invasive, does not require anesthesia, and has good soft tissue contrast without contrast agents [6, 7]. Compared to caliper measurements, ultrasound requires more extensive operator training, but all three axes of the tumor can be easily measured, relaxing the assumption on the ellipsoid being axisymmetric. A comprehensive view of the tumor, eliminating the need for shape approximations, can further be achieved using 3D ultrasound imaging methods. Multiple studies have validated the reliability of 3D ultrasound imaging for volume assessment, citing its accuracy and reproducibility compared to caliper measurements [7–14]. Despite these advantages, 3D ultrasound is not without its drawbacks; it necessitates specialized equipment and software, and the imaging process can be time-consuming, which in turn introduces anesthesia and heating considerations.

Conventional 2D ultrasound imaging in contrast offers short scan times without the need for anesthesia or heating, favorable factors in longitudinal studies on large animal cohorts from ethical and logistical perspectives. The accuracy of this 2D technique which requires approximations to be made on the tumor shape has not been studied as thoroughly as 3D imaging. To the best of our knowledge, a recent publication by Molière et al. [15] is the only relevant study in this area. This study involved tumors were implanted in the mammillary gland in immunocompetent mice, resulting in a mixture of growing and regressing tumors. Using excised tumor volumes as reference, they found that 2D ultrasound imaging could facilitate precise and efficient tumor evaluation in preclinical settings. However, assessment of volume estimation by 2D ultrasound imaging radiotherapeutic and longitudinal settings remain unexplored. Furthermore, the comparative accuracy of caliper and ultrasound measurements has not been juxtaposed with magnetic resonance imaging (MRI)-based tumor volume estimation.

MRI is another method for depicting morphology in vivo. Unlike calipers and ultrasound, MRI can produce high resolution 3D images with accurate geometry and superior soft tissue contrast. Thereby, it enables accurate tumor volume measurements that do not rely on any assumptions on its shape, which can be used as a non-invasive gold standard. However, MRI has limitations for longitudinal tumor volume estimation studies, such as being expensive and requiring long preparation and acquisition times during which the animal must be anesthetized.

In this study, we evaluate the accuracy of tumor volume estimation by caliper and 2D ultrasound, comparing the results to reference measurements obtained by MRI. Our objective was to replicate a typical therapeutic study structure, which encompassed two distinct treatment modalities and a control group. The study was longitudinal and performed on nude mice that were subcutaneously inoculated with a human prostate cancer cell line, including groups receiving no treatment, external beam radiotherapy, or radionuclide therapy. We show that caliper measurements have a marked positive bias (for example including skin and surrounding tissue in tumor tissue measurements) and that ultrasound has superior accuracy.

## Materials and methods

### In vivo prostate cancer tumor model

We used BALB/c^nu/nu^ (Janvier, Le Genest-Saint-Isle, France) nude mice for the therapy studies. We implanted LNCaP cells (5–7 ×10^6^ cells per mouse) (Prostate Carcinoma cells Clone FGC ATCC®CRL-1740, Lot 5972254) subcutaneously in a 200 μL cell suspension (1:1 mixture of Matrigel, Corning and RPMI 1640 medium) on the right hind leg 4–6 weeks prior to the experiment. All animal experiments were performed in accordance with national legislation on laboratory animal protection and permitted by the local ethics committee for animal research at Lund University (permit number 4350–20). The animals were regularly monitored for tumor growth and body weight during the full time of the study once a week. All animals were housed under controlled conditions with free access to water and standard rodent chow and physical signs of illness were monitored 3 times a week. The cages were equipped with cardboard tunnels and shavings. If a weight loss of 20% or if a severe decline in general condition (ex. ruffled fur, paralysis) or if the tumor diameter reached > 15 mm or a volume of over 1000 mm^3^ was noticed, animals were immediately euthanized. No animals died before meeting the criteria for euthanasia. Euthanasia was performed under CO2 gas inhalation, and all efforts were made to minimize suffering. A total of 29 mice were included in the study and assigned to three groups: one group (n = 5) was treated with EBRT, one group (n = 3) was treated with [^177^Lu]Lu-PSMA-617, and one group (n = 21) was not treated (control). The total duration of the therapy experiments was up to 50 days.

### Radiotherapy and control groups

External beam radiotherapy was performed at the XenX small animal irradiation platform (XStrahl Inc, Suwanee, GA, USA) with a beam energy of 220 kV and a 0.15 mm Cu filter, calibrated in accordance with the IAEA TRS-398 reference dosimetry protocol [16]. During irradiation, the animals were placed in a ventilated tube located in the treatment position using laser pointers and anesthetized with a continuous administration of isoflurane. A preparatory treatment plan was produced in μRaystation (RaySearch Laboratories AB, Stockholm, Sweden) to ensure full target coverage using a single beam with an adjustable beam collimator while sparing as much as possible of the surrounding normal tissue. The absorbed dose to the target was 10 or 20 Gy delivered in a single treatment fraction with a dose rate of 2.8 Gy/min at a source-to-skin distance of 35 cm. The delivered dose was verified using GafChromic EBT3 film (Ashland Advanced Materials, Bridgewater, NJ, USA). The entire procedure was completed within about 10 minutes and the animals were returned to their cages after the treatment session.

Mice in the radionuclide therapy group were injected through the tail vein with 15–17 MBq of [^177^Lu]Lu-PSMA-617. For labeling of PSMA please see S1 Appendix. Mice were euthanized 5 weeks after radiotherapy.

One group of mice inoculated with LNCaP tumors was left untreated. The first tumor volume measurement was performed 5–6 weeks after inoculation.

### Longitudinal volume estimation

The tumors in each group were measured at several timepoints before and after treatment using a digital caliper, ultrasound, and MRI as follows.

### Caliper measurements

The tumor size was measured by a researcher familiar with the method (MS) with 2 years’ experience of tumor measurements using calipers. To minimize variability, all measurements were performed under similar conditions. The tumor volume was estimated by measuring the length (*l*) and width (*w*) of the tumors, and the volume (*v*) was calculated as the volume of an axisymmetric ellipsoid (*w* = *d*) such that the volume $v_{Caliper}=\frac{\pi }{6}l\cdot w\cdot d\approx \frac{1}{2}l\cdot w^{2}$ [17].

### Ultrasound measurements

Ultrasound images were acquired for each tumor using an 8–20 MHz portable handheld scanner (Clarius Mobile Health, LD20HD), by a researcher with adequate training. During image acquisition, mice were awake, and tumors were covered in ultrasound gel. The ultrasound probe was positioned lightly without pressure over the tumor perpendicular to the surface. By rotating the probe, images of tumors were taken in the transversal and coronal plane. For analysis of ultrasound data, images were imported into a program supplied by the manufacturer. The tumors were well defined in the images. The length (*l*), width (*w*) and depth (*d*) were measured from the borders of each tumor using an integrated measuring tool in the program. The depth of the tumor was measured twice; in both the coronal and the transversal planes. To obtain a representative value, an average of the two measurements was calculated. To calculate the volume of the tumor, its shape is assumed to be approximately ellipsoidal with a volume of *v*_US_ = *l*∙*w*∙*d*/2.

Further details on volume estimation from caliper and ultrasound measurements are provided in S1 Appendix.

### MRI measurements

The mice were examined by MRI at a 9.4 T (Bruker, BioSpec Avance III) with an 86 mm quadrature transmit coil and 10 mm or 20 mm linear surface receive coils depending on the size of the tumor. During imaging the animals were anesthetized with a continuous administration of isoflurane. Respiration and temperature were continuously monitored during imaging.

The tumor morphology was depicted by rapid acquisition with relaxation enhancement (RARE) [18] using acceleration factor = 8, number of averages = 6–8, effective TE = 25–30 ms, TR = 2.0–2.5 s, in-plane resolution = 0.125×0.125 mm^2^, slice thickness = 0.5–1.0 mm, and field-of-view = 20×20×10 mm^3^, for a total acquisition time of approximately 5 min. Note that the parameters were not identical for all acquisitions, but we expect this to have a negligible effect on the tumor volume estimation.

Tumor regions of interest (ROIs) were manually defined in the RARE images, and volumes were calculated as *v*_MRI_ = *n*_voxels_∙*n*_voxel_, where *n*_voxels_ is the number of voxels in a ROI and *v*_voxel_ is the volume of a single voxel. The ROIs were delineated by an expert researcher in MRI imaging.

### Analysis of accuracy in volume estimation

Paired volume estimations (same subject and time, but different methods) were used to establish the accuracy of caliper and ultrasound using MRI as a reference. Since not all methods were used at all timepoints (see Fig 3), an imputation procedure was used to fill in missing data by interpolation. Briefly, for each mouse and a given method-pair, we identified the overlapping time-interval where both methods were used. For all days within this interval where only one of the methods was used, the data point for the other method was imputed by linear interpolation based on the neighboring data points [19].

Evaluations and comparisons were made between the performance of volume estimation using caliper and ultrasound. With pairs of data-points (volume estimates of the same tumor and date), the accuracy of both methods was studied using the MRI-estimated volumes as reference. Since the data distribution appeared to be non-normal according to Shapiro-Wilk tests, we used two-sided Wilcoxon tests (paired, non-parametric) to test if the medians of the volume errors differed from zero using SciPy [20]. A significance level of 0.05 was used as threshold for statistical significance. The volume errors were also expressed as relative differences (*E*_rel_), calculated as

$$E_{rel}=\frac{v_{x}-v_{MRI}}{v_{MRI}},$$ (1)

where *v*_*x*_ denotes a volume estimated by ultrasound or caliper, and *v*_MRI_ denotes the corresponding estimate by MRI.

Since comparisons that involved volumes measured by MRI reduced the number of available data points, the caliper and ultrasound methods were also compared separately by studying the estimated volumes for paired data involving only these methods. Whether these two methods produced similar volume estimations was tested for using a paired two-sided Wilcoxon test, with a significance threshold of 0.05. A second purpose of this test was to indirectly obtain information about differences in volume errors between caliper and ultrasound, as $(v_{Caliper}-v_{MRI})-(v_{US}-v_{MRI})=v_{Caliper}-v_{US}$, i.e. a difference in volume error between two methods with a third method as reference is equivalent to a difference in volume between the two former methods. Data imputation was not needed for this test since all days with ultrasound imaging included caliper measurements.

## Results

### Tumor volume measurements

**Fig 1:**
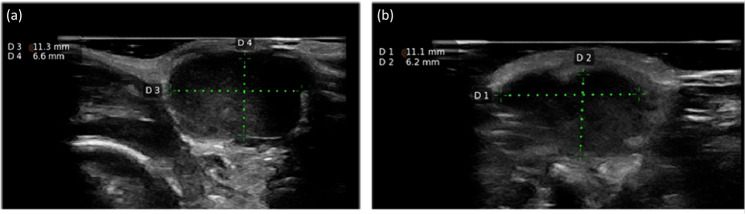
An example of an ultrasound image obtained using the portable handheld ultrasound probe of a LNCaP tumor inoculated in in the right flank of a nude mouse. The image was obtained 37 days after inoculations. The tumor is seen as a hypoechoic mass. The figure shows a transversal view of the tumor measuring 11.3 mm in width and 6.6 mm in depth (a) and a coronal view of the tumor measuring 11.1 mm in length and 6.2 mm in depth (b).

Measurements using ultrasound were straightforward; each typically lasted less than 2 minutes and there was no need to anesthetize mice to obtain sufficient image quality. The tumors were well defined in the images and the tumor borders had a high contrast to surrounding tissue, an example of an ultrasound image is presented in Fig 1.

**Fig 2:**
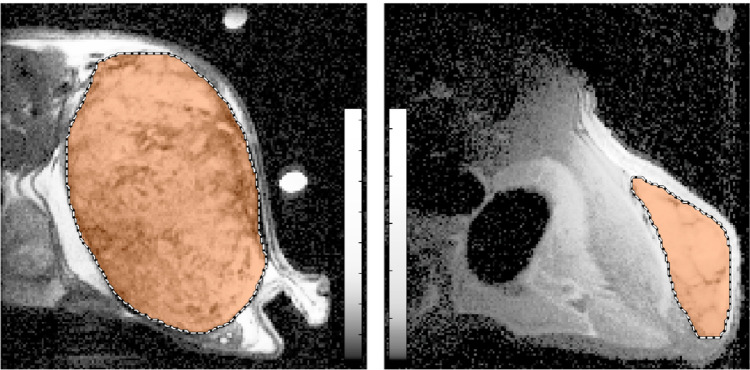
Examples of MR images and ROIs for a large tumor (left, 1.3 cm^3^) and a small tumor (right, 0.06 cm^3^). MR images are shown with histogram-equalized colormaps, and ROIs are shown as orange regions with black and white dashed borders. The large tumor is well-approximated by an ellipsoid shape, and the small tumor is less accurately represented, although this is difficult to convey in two-dimensional images.

**Fig 3:**
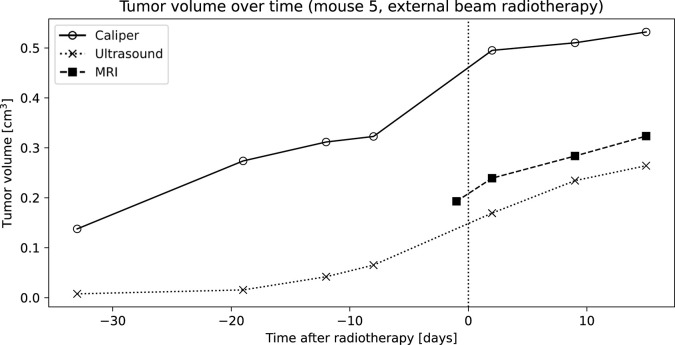
Example of tumor volumes measured with caliper, ultrasound, and MRI in one mouse treated with EBRT. The plot illustrates the data available for the method comparison, wherein growing tumors are assessed by each of the three devices, albeit at different time points. Data imputation inside the overlapping time intervals facilitated thorough comparisons on this dataset.

Measurement of tumor volume using MRI was successful in all cases, and tumor ROIs were straightforwardly defined following the clear boundary between tumor and surrounding tissue (Fig 2). Examples of tumor volumes measured in one mouse are presented in Fig 3.

**Fig 4:**
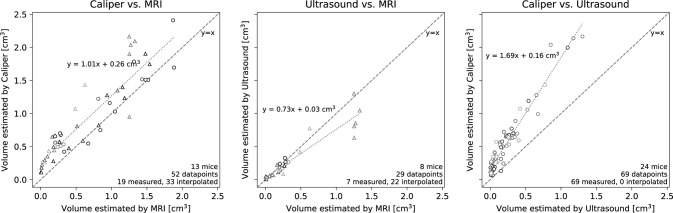
Comparisons of estimated tumor volumes between pairs of methods. Data points where volumes were estimated by both methods are shown as circles whereas data where one point was estimated by linear interpolation is shown as triangles. Data derived from each mouse is color coded consistently across plots. Linear regression lines are shown as dotted lines, and the identity line is shown as a broken gray line. The dotted lines show linear regression lines, with Pearson correlation coefficients of *r* = 0.91, *p*<10^−20^ (caliper-MRI), *r* = 0.94, *p*<10^−13^ (ultrasound-MRI), and *r* = 0.97, *p*<10^−40^ (caliper-ultrasound). The pairwise method comparisons demonstrate that caliper tend to produce greater volume estimations compared to both ultrasound and MRI, whereas the ultrasound and MRI methods are more consistent.

The volumes estimated by caliper measurements tend to overestimate the ultrasound and MRI estimates, whereas ultrasound and MRI show a better agreement (Fig 4).

**Fig 5:**
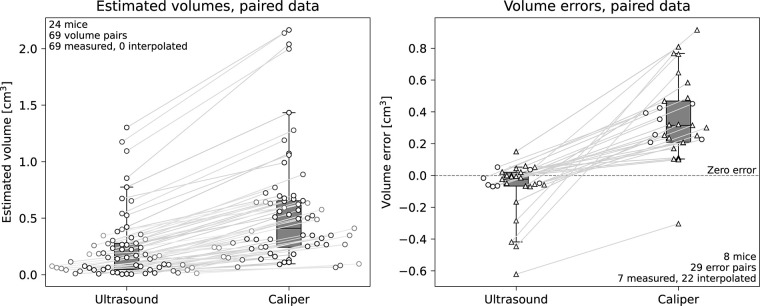
Distributions of paired volume estimates for paired ultrasound and caliper data (left) and distributions of volume errors in paired ultrasound and caliper measurements compared to MRI as reference (right). Data is visualized as box plots and the paired data-points are shown as scatter plots connected by lines. Whiskers indicate the 5^th^ and 95^th^ percentiles. Data involving only measured volumes are indicated by circles, and triangles indicate points produced by interpolation. The plot on the left shows that caliper measurements systematically yield larger volumes than those performed with ultrasound. The figure on the right shows that volume estimation by ultrasound is markedly more accurate than caliper. Indeed, caliper measurements overestimate the tumor volume by 0.21 to 0.47 cm^3^ in 50% of cases.

Fig 5 shows the distributions in volumes (*n* = 69, from 24 mice) and volume errors (*n* = 29, from 8 mice) for paired caliper and ultrasound data. The volumes measured by ultrasound are generally more accurate, with a median and inter-quartile range (IQR) in error of -0.02 cm^3^ (0.09 cm^3^), whereas corresponding values for measurements by caliper are 0.32 cm^3^ (0.26 cm^3^). The median volume errors for both ultrasound and caliper are significantly different from zero (Wilcoxon tests), with *p* = 0.019 and *p*<10^−6^ respectively. Expressed as relative differences (Eq 1), the median (and IQR) errors are –21% (44%) for ultrasound and 150% (190%) for caliper. The paired volume data shows that estimation by caliper exceeds those estimated by ultrasound in all but one data point. The median (and IQR) for the estimated volumes in Fig 5 is 0.15 cm^3^ (0.23 cm^3^) for ultrasound, and 0.41 cm^3^ (0.41 cm^3^) for caliper. A Wilcoxon test shows a significant difference in volumes estimated by the methods (*p*<10^−12^).

**Fig 6:**
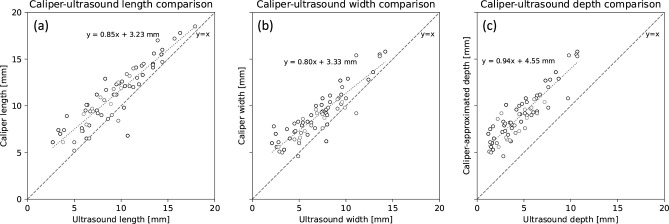
Panel a and b: Comparison of paired tumor lengths (a) and widths (b) measured by caliper and ultrasound. Panel c: Comparison of caliper-approximated tumor depths (i.e., widths) against tumor depths measured by ultrasound. Linear regressions are shown as dotted lines, with Pearson correlation coefficients of *r* = 0.91, *p* = <10^−26^ (a), *r* = 0.89, *p*<10^−24^ (b), *r* = 0.90, and *p*<10^−24^ (c). Length measurements by caliper exceed corresponding ultrasound measurements in a large majority of cases.

**Fig 7:**
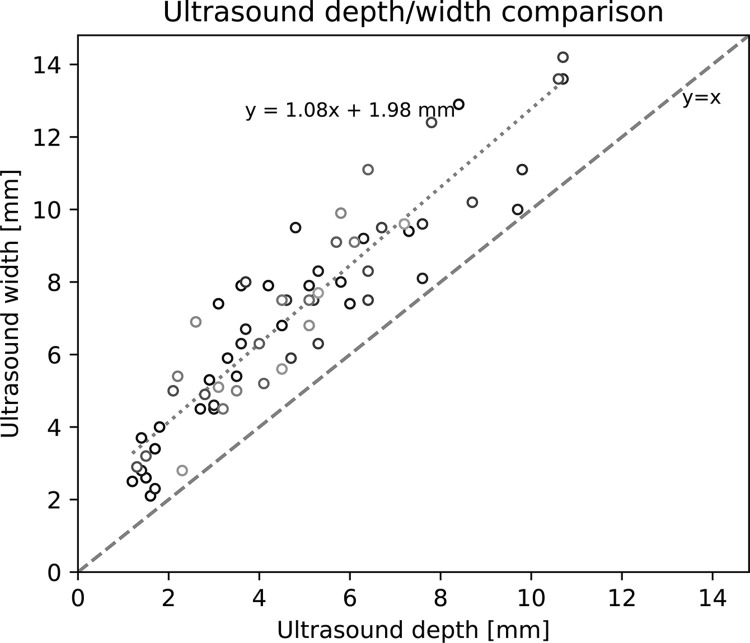
Comparison of tumor widths and depths measured by ultrasound. Linear regression is shown as a dotted line with a Pearson correlation coefficient of *r* = 0.92, *p*<10^−28^. Measurements in the ultrasound images finds that all tumors are slightly wider than they are deep, indicating that spheroid approximations may yield inaccuracies.

Fig 6 presents an investigation into potential causes for the caliper method’s tendency to overestimate tumor volumes, by comparing the length-, width-, and depth-axis measurements within the frameworks of ellipsoidal and spheroidal tumor approximations. The comparisons in paired lengths and widths (panel a and b) show that the caliper method tends to yield greater measurement values for both axes. When comparing the caliper method’s approximation for depth (i.e., width) against the depth measured by ultrasound (panel c), even larger differences are obtained. The validity of the axisymmetric ellipsoid tumor approximation (depth and width are equal) is investigated in Fig 7, which shows that the tumor depths are shorter than the widths in the ultrasound images. Taken together, Figs 6 and 7 indicate that the systematic overestimation in volumes measured by caliper (Fig 5) stems from both an overall tendency to overestimate all axis lengths as well as inaccuracies in the ellipsoid shape approximation.

## Discussion

Accurate tumor volume measurements are essential for evaluating the therapeutic response of radiopharmaceuticals and radiotherapy. Precise tumor volume measurements can improve the reproducibility, interpretation, and sensitivity of research results, as well as reduce the number of animals used in pharmaceutical development, which would lower the cost and increase the animal welfare. Moreover, inaccuracies in measured tumor volumes can affect the estimated absorbed doses in radionuclide therapy [4, 5], which may influence the statistical power of dose-response relationship studies. Therefore, accurate tumor volume measurements are crucial for the advancement of radiopharmaceutical therapy. Caliper measurements of tumor volume are commonly used to evaluate the therapeutic efficacy of radiotherapy and experimental drugs in pre-clinical models of subcutaneous xenografts [11, 17, 21]. However, this method has several drawbacks. Caliper measurements are based on imprecise estimation of the tumor dimensions and assume that the tumor has a spheroid shape with depth equal to its width [17]. However, tumors are (i) not spheroidal, and (ii) their width can be an inaccurate stand-in for their depth [2]. Both shortcomings lead to inaccurate volume estimation. Fig 2 illustrates a selection of MR images and ROIs, where this assumption is valid to varying degrees. In this study, we have shown that caliper measurements had a median volume error of 0.32 cm^3^ (150%) compared to –0.02 cm^3^ (-21%) for ultrasound measurements.

Tumor volume measurements by ultrasound have been investigated previously, e.g., [7, 8, 11, 13, 15, 22], although most evaluations involve 3D ultrasound imaging; a study by Molière et al. [15] being a recent exception that also evaluates volume estimation by 2D ultrasound. Compared to the portable 2D imaging system evaluated herein, 3D imaging involves more complex hardware and software and requires further heating and anesthesia considerations. Our results affirm ultrasound imaging in two dimensions as a useful method, and the availability of 2D and 3D modalities represent options in terms of speed and simplicity against comprehensive tumor depictions, with the latter alternative avoiding the need for volume approximations.

Fig 4 illustrates that the caliper method gives volume estimates that tend to exceed corresponding estimates by ultrasound and MRI. When studying volumes estimated by ultrasound and caliper against MRI (Fig 5 right), the ultrasound method is markedly more accurate. The small negative bias for ultrasound and larger positive bias for caliper in Fig 5 right is in-line with findings by Ayers et al [7] and Molière et al. [15]. The volumes were shown to be significantly greater when estimated caliper compared to ultrasound (Fig 5 left), which implies that these methods would also differ significantly in terms of volume errors. The tests on the volume-errors (i.e., using MRI as the reference) show that both the caliper and ultrasound methods have median errors significantly different from zero. However, we note that the 5^th^-95^th^ percentile intervals for the errors were [-0.4, 0.06] cm^3^ for ultrasound and [0.1, 0.8] cm^3^ for caliper, establishing ultrasound as a vastly more precise and accurate method. The tendency for caliper to produce positive biases when compared with other methods has also been noted by other research groups [7, 11, 13, 15].

Figs 6 and 7 reveal two likely explanations for why caliper measurements consistently overestimate the volume. First, the caliper method tends to overestimate both the length and width of the tumors (Fig 6). Secondly, the assumption of equal width and depth appears to be incorrect such that the tumor width tends to be an overestimation of its depth (Fig 7), in-line with findings by Brough et al. [2]. Finally, we note that it is the estimation of the length, width and depth of the tumor that fails when using caliper-based volume estimation, rather than an inconsistency in the assumption of an ellipsoidal shape. Indeed, ultrasound measurements based on this assumption were quite accurate for the xenografts investigated herein (Figs 4 center and 5 right). Further we also show that the width and length estimations using caliper is most inaccurate in small and medium sized tumor volumes which are often of most interest in preclinical therapy studies, and that the error seem to decrease with increasing tumor size. This could possibly be explained by tightening of the skin, easier to measure, and more pronounced tumor. This could affect the assessment of tumor growth and thus assessment of therapy efficacy.

Studies investigating the performance of volume estimation by 2D ultrasound imaging are limited. A recent publication by Molière et al. [15] is the only other relevant study in this area we are aware of, also notes the prevalence of 3D over 2D imaging. Although 3D imaging is more comprehensive and likely more accurate for volume estimation, it is more time-consuming and places further demands on anesthesia and heating—important factors in longitudinal studies where tumor volumes may be monitored in large animal cohorts with high frequency over long time-periods. The results presented herein, along with those by Molière et al. [15], highlight 2D ultrasound imaging as a favorable modality for longitudinal studies. Taken together these two studies demonstrate good performance of 2D ultrasound in a wider range of applications: whereas our study includes large (up to about 1.5 cm^3^) mostly growing prostate cancer tumors in immunoincompetent mice in a radiotherapy setting, the other study investigates a mixture of growing and regressing small (up to 0.16 cm^3^) breast cancer tumors in immunocompetent mice.

The boundary between tumor and normal tissue in the MR-images were in some instances ambiguous, and the drawn ROIs have not been validated against other methods such as whole-mount histology. Thus, some degree of uncertainty and error must be attributed to the volumes attained from MRI. However, since MRI offers excellent soft tissue contrast and enables viewing and delineation of the entire tumor volume in three dimensions without any approximations, we still consider MRI to be highly qualified as a reference method.

Computed tomography (CT) imaging is an alternative to caliper measurements for tumor volume estimation. Just like MRI, it has the advantage of producing three-dimensional images at high spatial resolution. CT requires that the animals are anesthetized during the acquisition which can induce stress and potentially affect the experimental results [23, 24]. Ultrasound imaging has several advantages over CT when determining the tumor volume, such as availability, ease of learning, fast acquisition time and no need for anesthesia.

A summary and comparison of the methods used in this study is presented in Table 1. The results obtained herein indicate that ultrasound can be a practical and suitable method for subcutaneous tumor volume measurements with a good compromise of accuracy, availability, and price. In the present study we only used one trained person for caliper and ultrasound measurements, it would be interesting to evaluate if the measurements errors are the same between different operators and different measurement techniques (e.g., pushing in the skin or barely touching it). An additional topic of interest for future research would be to explore the performance of 2D ultrasound imaging for volume estimation of non-superficial tumors. As such tumors may exhibit more irregular growth patterns compared to superficial xenografts and may thus challenge the ellipsoidal shape approximation [15], such a task has been considered out of scope for this study.

**Table 1:** Summary of the strengths and weaknesses of the investigated methods. When compared to caliper and MRI, 2D ultrasound offers a reasonable balance between cost, throughput, and volume accuracy.

Method	Cost	Measures tumor depth	Handling and throughput	Volume accuracy	Requires anesthesia	Additional factors
**Caliper**	$	No	+ + +	+	No	Affected by skin and fur
**Ultrasound**	$ $ $	Yes	+ +	+ + +	No	Imaging^a^
**MRI**	$ $ $ $ $	Yes	+	+ + + +	Yes	Imaging^a^

^a^Imaging methods can provide additional data on the tumor beyond its volume, by e.g. revealing necrotic regions or measuring micro-structure parameters.

## Conclusions

In this study, we have assessed the accuracy of volume estimation in sub-cutaneous tumors by standard calipers and ultrasound imaging in mice undergoing external beam radiotherapy, radionuclide therapy, as well as an untreated control group. Using MRI as a reference, the ultrasound method was found to be more accurate than external calipers for volume estimation. By presenting a good balance of affordability, throughput, and accuracy, the ultrasound system is found to be favorable for tumor volume assessment in longitudinal pre-clinical studies. For studies where the tumor volume is used to assess treatment-outcome or for dosimetry calculations, the superior accuracy of ultrasound can support more accurate dose response analysis and may provide a means of reducing the necessary sample size.

## Supporting information

S1 Appendix 1: Ellipsoidal approximations for volume estimation and 2: Labelling chemistry. (PDF)

S1 Dataset Spreadsheet with tumor dimensions and volumes measured with caliper, ultrasound, and MRI. (XLSX)

## List of abbreviations

CT: Computed tomography
EBRT: External beam radiotherapy
IQR: Inter-quartile range
MIRD: Medical internal radiation dose
MRI: Magnetic resonance imaging
RARE: Rapid acquisition with relaxation enhancement
ROI: Region of interest
