# Supplementary material for: Evaluation of superficial xenograft volume estimation by ultrasound and caliper against MRI in a longitudinal pre-clinical radiotherapeutic setting
Source: PLoS One. 2024 Jul 25;19(7):e0307558. doi: 10.1371/journal.pone.0307558 (PMC11271909; doi:10.1371/journal.pone.0307558)
Supplement: S1 Appendix — (PDF) [file pone.0307558.s001.pdf]

Appendix

1: Ellipsoidal approximations for volume estimation

An ellipsoid region can be described by the constraint

$$\frac{x^2}{a^2} + \frac{y^2}{b^2} + \frac{z^2}{c^2} \leq 1, \tag{S1}$$

where (x, y, z) are Cartesian coordinates and a, b, and c are the lengths of the ellipsoid's semi-axes (analogous to sphere radius when $a = b = c$). To simplify the estimation of the volume of a region, it can be approximated as an ellipsoid whereby a volume (v) can be calculated from measurements on the three semi-axes. The volume for the region described by Eq. S1 is

$$v = \frac{4\pi abc}{3}. \tag{S2}$$

Often it is more practical to measure the full length of the ellipsoid along its semi-axes, $l = 2a$, $w = 2b$, $d = 2c$, for which Eq. S2 becomes

$$v = \frac{\pi lwd}{6}. \tag{S3}$$

For a tumor xenograft, the three full lengths can be considered as the tumor length (l), width (w), and depth (d). As the assumption of an ellipsoidal tumor is only an approximation, Eq. S3 can be simplified further by approximating $\pi \approx 3$:

$$v = l \cdot w \cdot d/2. \tag{S4}$$

The depth-axis of a tumor xenograft is generally hard to assess with caliper. To enable volume estimation in this situation, the tumor can be approximated further as a spheroid where the depth-axis and width-axis have equal lengths, $d = w$. This yields

$$v = l \cdot w^2/2. \tag{S5}$$

Errors in estimated volume may arise if the simplifying assumptions are not met. E.g., if the region does not resemble an ellipsoid or spheroid. Volume errors may also arise if the measured axes aren't perpendicular, or don't align with the actual semi-axes. For irregularly shaped regions, full three-dimensional tomographic imaging is likely to produce more accurate volume estimates.

2: Labelling chemistry

Following a previously published protocol [25] approximately 100 µL of sterile sodium acetate solution (0.4 M, pH 5.5) and 1.25 µL ascorbic acid solution (20 % w/w) were added to circa 2 – 3 µL (60 – 75 MBq in total) of non-carrier added ^{177}Lu (ITM Isotope Technologies Munich, Garching, Germany). To this solution, 60 nmol (volume circa 30 µL) of PSMA-617 (MedChemExpress, Monmouth Junction, NJ, USA) were added and labeled by incubating on a shaker at 95 °C for 15 min. The reaction was terminated by cooling to room temperature. At 0 min and 15 min, respectively, 1 µL of the solution was added to an instant thin layer chromatography (iTLC) strip. As a mobile phase, sodium citrate

solution (0.2 M, pH 2) was used, and the percentage of free ^{177}Lu was determined by analyzing the iTLC strips with a phosphor imager system (Cyclone Plus Phosphor Imager, PerkinElmer, Inc., Waltham, MA, USA). Thereafter, a sterile 0.9 % sodium chloride solution was added. A small sample for iTLC was taken, and pH tested before injections in mice. The radiochemical purity of the radioligand was >99 %. [^{177}Lu]Lu-PSMA-617 was labeled at a specific activity of 62 MBq nmol^{-1} (judged most effective for therapy by Fendler et al. [26]).

References

[25] Kristiansson A, Vilhelmsson Timmermand O, Altai M, Strand J, Strand SE, Akerstrom B, et al. Hematological Toxicity in Mice after High Activity Injections of (177)Lu-PSMA-617. Pharmaceutics. 2022;14(4).

[26] Fendler WP, Stuparu AD, Evans-Axelsson S, Luckerath K, Wei L, Kim W, et al. Establishing (177)Lu-PSMA-617 Radioligand Therapy in a Syngeneic Model of Murine Prostate Cancer. J Nucl Med. 2017;58(11):1786-92.
